# Dyslipidemia and Associated Risk Factors in the Elderly Population in Asmara, Eritrea: Results from a Community-Based Cross-Sectional Study

**DOI:** 10.1155/2021/6155304

**Published:** 2021-09-30

**Authors:** Oliver Okoth Achila, Mathewos Araya, Arsiema Brhane Berhe, Niat Habteab Haile, Luwam Kahsai Tsige, Bethelihem Yemane Shifare, Tesfalem Abel Bitew, Israel Eyob Berhe, Samuel Tekle Mengistu, Eyob Garoy Yohaness

**Affiliations:** ^1^Department of Clinical Laboratory Sciences, Orotta College of Medicine and Health Sciences, Eritrea; ^2^Asmara College of Health Sciences, Eritrea; ^3^Nakfa Hospital, Eritrea

## Abstract

**Background:**

The ultimate goal of the study was to approximate the burden and patterns of dyslipidemia in a subset of the elderly population (≥60–85 years) living in Asmara, Eritrea, and to identify modifiable risk drivers.

**Methods:**

A total of 319 (145 (45.5%) male vs. 174 (54.5%) female, mean age ± SD (68.06 ± 6.16 years), participants from randomly selected estates within Asmara were enrolled. Demographic and medical information was collected using a standardized questionnaire. Anthropometric, lipid panel, fasting plasma glucose (FPG), and blood pressure (BP) measurements were subsequently taken.

**Results:**

The prevalence of dyslipidemia was 70.5%. The proportions of dyslipidemias were (in order of decreasing frequency) high TC (51.2%), LDL-C (43.7%), low HDL-C (28.2%), and TG (27.6%). The average (±SD) concentrations in mg/dL of TC, LDL-C, non-HDL-C, TG, HDL-C, TC/HDL-C, and TG/HDL-C were 202.2 ± 40.63, 125.95 ± 33.16, 151.72 ± 37.19, 129 ± 57.16, 50.48 ± 10.91, 4.11 ± 0.91, and 2.72 ± 1.49, respectively. Furthermore, 17.5%, 21.6%, 11.0%, and 5.0% had abnormalities in 1, 2, 3, and 4 lipid disorders with the copresence of TC+LDL-C abnormalities dominating. Regarding National Cholesterol Education Program Third Adult Treatment Panel risk strata, 18.5%, 14.5%, 28.2%, and 12.9% were in high or very high-risk categories for TC, LDL-C, TG, and HDL-C, respectively. The high burden of dyslipidemia coexisted with an equally high burden of abdominal obesity (43.1%), FPG ≥ 100 mg/dL (16%), hypertension (28.5%), and physical inactivity. Overall, dyslipidemia was associated with sex (females: aOR = 2.6, 95%CI = 1.1–6.1, *p* = 0.017) and daily physical activity—higher in individuals undertaking physical activity for <1 hour (aOR = 2.6, 95%CI = 1.1–6.1, *p* = 0.029), 1-2 hours (aOR = 3.2, 95%CI = 1.24–8.5, *p* = 0.016), and 2-3 hours (aOR = 2.0, 95%CI = 0.7–5.8, *p* = 0.192) (Ref: >3 hours). Additional associations included increasing FPG (aOR = 1.02, 95%CI = 1.0–1.04, *p* = 0.039), and BMI (aOR = 1.19, 95%CI = 1.09–1.3, *p* < 0.001). These factors, along with waist circumference (WC), consumption of traditional foods, systolic BP, and diastolic BP, were, with some variations, associated with disparate dyslipidemias.

**Conclusions:**

The burden of dyslipidemia in the elderly population in Asmara is high. Modifiable risk drivers included FPG, WC, physical inactivity, and low consumption of traditional food. Overall, efforts directed at scaling up early recognition and treatment, including optimal pharmacological and nonpharmacological therapy, at all levels of care, should be instituted.

## 1. Introduction

Globally, the proportion of people > 60 years (elderly) is expected to double between 2000 and 2050 with the elderly outnumbering the young (<5 years old) in many countries in the near term [1]. This represents a formidable problem for low- and middle-income countries (LMIC) since this subgroup is inherently vulnerable to cardiovascular diseases (CVDs) [2] and is susceptible to multimorbidity, malignancies, neurodegenerative diseases, polypharmacy, and long disability periods, among others. For example, the aging and growth of the populations across the world resulted in an increase in global CVD-related mortality between 1990 and 2013, despite a decrease in age-specific death rates in most regions. More recently, the 2019 global burden of disease (GBD) estimate reported that CVDs caused approximately 17.8 million deaths globally [3] with a disproportionate number of cases in LMIC—a significant proportion of these cases were in individuals > 60 years, with an even larger prevalence (>85%) in individuals > 80 years in some jurisdictions [4]. Therefore, it can be argued that increases in years of life lost (YLLs), years lived with disability (YLDs), and losses in disability-adjusted life years (DALY) associated with heart failure (HF), coronary heart disease (CHD), peripheral arterial disease (PAD), and atrial fibrillation (AF) [2] epitomize the convergence of CVDs and aging in a rapidly aging world.

From an epidemiological standpoint, aging populations in Sub-Saharan Africa (SSA) will continue, at least partially, to shift the long-standing paradigm of infectious disease dominance to a multifaceted scenario where aging and NCDs, particularly CVDs, are playing an increasingly important role [5]. Predictably, few countries in SSA can shoulder the disproportionate number of hospitalizations, procedures, and costs associated with the emerging burden of CVDs and other age-related disorders in their elderly populations. Besides age, which is regarded as a CVD risk equivalent (most individuals are already at (very) high risk at the age of 65 years) [6], available evidence indicates that multiple modifiable risk factors can trigger or augment the said risk [7]. A 2013 GBD project estimation model indicated that the population-attributable fraction (PAF) for individual risk factors for CHD burden in LMICs was as follows: high blood pressure, 54%; high cholesterol concentrations, 32%; body mass index ≥ 25 kg/m^2^, 18%; unhealthy diet, 67%; and smoking, 18% [8]. In other words, the report indicated that dyslipidemia is one of the leading contributors to CVD and mortality in elderly populations across the world.

The absolute risk associated with pathological changes in lipids rises substantially with advancing age [2]. For instance, the Established Populations for Epidemiology Studies in the Elderly (EPESE) study noted that elevation in total cholesterol (TC), low-density lipoprotein cholesterol (LDL-C), and high-density lipoprotein cholesterol (HDL-C) concentrations is associated with increased morbimortality in older adults. Other investigators have demonstrated a linear, dose-dependent relationship between total cholesterol (TC) and CHD without an obvious lower threshold of TC [9]. Crucially, and taking the controversies regarding the management of dyslipidemias in geriatrics (particularly the mass use of statins in centenarians) into account, the Prospective Study of Pravastatin in Elderly at Risk (PROSPER) and Intervention Trial Evaluating Rosuvastatin (JUPITER) established the efficacy and safety of statin therapy in the reduction of primary composite endpoints of coronary death, nonfatal myocardial infarction (MI), and cerebrovascular accident (stroke) in the elderly [10, 11]. More to the point, the data suggests that the proper management of dyslipidemia can substantially attenuate CVD risk or morbimortality in a significant proportion of the elderly population [12, 13].

Even though dyslipidemia is regarded as one of the most important CVD risk factors in the elderly [2] and ranks highly in prominent CVD prevention guidelines, the condition is underdiagnosed and undertreated in elderly subgroups in SSA. Additionally, critical knowledge gaps on prevalence, pattern, and distribution of key risk factors remain. These descriptions describe the situation in Eritrea where a growing burden of CVD [14] (due to increasing life expectancy, among others) is compounded by suboptimal treatment, lack of knowledge and awareness, and limited understanding of the epidemiology of dyslipidemia. The present study was therefore undertaken to provide baseline data on dyslipidemia. Considering the underrepresentation of the elderly subgroups in dyslipidemia-related community-based surveys from SSA, we believe that this study provides significant information.

## 2. Methods

### 2.1. Study Setting and Study Design

This was a community-based cross-sectional study conducted among the elderly population (age ≥ 60–85 years) between January and June 2018 in Asmara, Eritrea.

### 2.2. Sample Size Calculation, Participant Recruitment, and Selection

The study targeted the elderly civilian population in Asmara, Eritrea. The choice of the setting was informed by the fact that Asmara has a relatively large population of elderly citizens. It is also regarded as the most cosmopolitan center in the country owing to its status as the capital city. The sample size was estimated using a single proportion formula. The prevalence of dyslipidemia was assumed to be 30% [15], with a margin of 5% and a 95% confidence interval (CI). After adjustments for nonresponse (10%), a total of 319 participants were recruited.

To locate participants, a stratified sampling design was employed. Based on previous experience, 13 estates (1 estate per subzone) were selected using the lottery method. Individuals ≥ 60 to ≤85 years working or residing within the selected estates were subsequently invited to participate in the study. Apart from age, additional inclusion criteria included Asmara city residents for at least 1 year and the ability to give consent. On the other hand, the exclusion criterion was based on the following considerations: mental illness/dementia, persons not willing to grant consent, and diabetes mellitus (DM) patients.

### 2.3. Data Collection, Measurements, and Definitions

#### 2.3.1. Demographic and Health History

A predesigned data collection form (the World Health Organization (WHO) STEPS questionnaire) [16] was used to collect data. Demographics, factors associated with lifestyle, medications (antilipid medications), and existing comorbidities were collected using this document.

#### 2.3.2. Clinical Biochemistry Measurements

Using established protocols (standardized posture—sitting quietly for 15 min before venipuncture and ≥8-hour fast), 5 mL of blood was drawn from the median cubital vein. The collected specimen was subsequently used for the analysis of the following markers using a Beckman Coulter AU480 Chemistry System: lipid panel markers—triacylglycerol (TG), total cholesterol (TC), high-density lipoprotein cholesterol (HDL-C), and fasting plasma glucose (FPG). The Friedewald formula (LDL = non − HDL − C − TG/5 (mg/dL)) was used to estimate LDL-C concentration for participants with TG level < 400 mg/dL. Non-high-density lipoprotein cholesterol (non-HDL-C) was calculated by subtracting HDL-C from TC. In addition, lipid ratios including TC/HDL-C ratio and TG/HDL-C were computed. National Cholesterol Education Program Third Adult Treatment Panel (NCEP-ATP III) guideline [17] was used to evaluate abnormalities in lipid panel markers. Dyslipidemia was defined as having at least one of the following: total cholesterol (TC) ≥ 200 mg/dL; triglycerides (TG) ≥ 150 mg/dL; low − density lipoprotein cholesterol (LDL − C) ≥ 130 mg/dL; and high − density lipoprotein cholesterol (HDL − C) ≤ 40 mg/dL for males and HDL − C ≤ 50 mg/dL for females. Mixed dyslipidemia was defined as the copresence of ≥2 dyslipidemias. The World Health Organization (WHO) cut-off point was used for the assignment of FPG status. Accordingly, normal FPG levels were defined as FPG ≤ 110 mg/dL, prediabetes (FPG ≥ 110–124.9 mg/dL), and potential diabetes mellitus (FPG > 125 mg/dL).

#### 2.3.3. Anthropometric Measurement

Relevant anthropometric information including height (Ht), weight, body mass index (BMI), and waist circumference (WC) (measured at the iliac crest) was collected as per established guidelines [18, 19]. For instance, height and weight were measured using a stadiometer (Hardik Meditech, India) and a bathroom scale (Zhongshan Camry Electronic Co. Ltd., China), respectively. BMI was then calculated by dividing weight (kg) by height (meters^2^). All measurements were taken in duplicate, and the average of the two measurements was presumed to be a better approximation of the true value. BMI groupings were defined as per WHO guidelines [19]: BMI ≤ 18.5 kg/m^2^ (underweight), 18.6-24.9 kg/m^2^ (normal weight), 25-29.9 kg/m^2^ (overweight), ≥30-34.9 kg/m^2^ (obese class I), 35-39.9 kg/m^2^ (obese class II), and ≥40 kg/m^2^ (morbid obesity). Due to the absence of a locally appropriate WC cut-off point, the International Diabetes Federation (IDF) specifications were used (WC > 94 cm (males)/80 cm (females)) [18].

#### 2.3.4. Blood Pressure (BP) and Hypertension Status

Blood pressure was measured using a calibrated digital sphygmomanometer (MDF® Lenus Digital Blood Pressure Monitor) and appropriately sized arm-cuff. Briefly, the participants were instructed to rest for at least 15 min. Three systolic and diastolic BP measurements were taken (at least 5 minutes apart). The last two measurements were then averaged and used in this study. Hypertension was defined as per the Joint National Committee on the Prevention, Detection, Evaluation, and Treatment of Hypertension (JNC-8) guidelines DBP/SBP ≥ 90/140 mmHg [20] or previous diagnosis of hypertension and being on antihypertension medication.

### 2.4. Data Analysis

Data analysis was conducted using IBM SPSS Statistics (SPSS Inc., Version 20.0, and Chicago, IL, USA). Where appropriate, percentages, mean ± SD, or median ± interquartile range (IQR) were used to describe the variables. Normal data distribution was evaluated using the Kolmogorov-Smirnov test, Shapiro-Wilk test, and visual inspection of Gaussian plots. Assessment of homogeneity of variances was undertaken using the Levene test. Depending on the data distribution of category, Student *t*-test or Mann–Whitney *U* test (numerical variables) and chi-square (*χ*^2^) test/or Fisher's exact test (categorical variables) were employed. Chi-squire trend test (linear-by-linear association) was reported where applicable. Binary logistic regression (backward: conditional) was undertaken to identify anthropometric, lifestyle, and clinical parameters independently associated with increased odds of having abnormal levels of TC, LDL-C, non-HDL-C, TG, HDL-C, TC/HDL, and TG/HDL and at least one dyslipidemia. Adjusted odds ratio (aOR) and 95% confidence interval (95% CI) values were subsequently reported. Two-sided *p* value < 0.05 was used to evaluate statistical significance.

## 3. Results

### 3.1. Demographic, Anthropometric, and Clinical Characteristics of Study Participants

The study sample comprised 319 (145 (45.5%) males vs. 174 (54.5%) females. The mean ± SD for age was 68.06 ± 6.16 years. Based on our interviews, we noted that none of the study participants were on antilipid medication, and knowledge about lipid abnormalities was marginal < 0.01%. Out of the total study population, 91(28.5%) had hypertension, 192 (60.2%) were in the 60-70-year age range, 268 (84.0%) had normal FPG, 32(10%) had impaired/prediabetes FPG, and 19 (6.0%) were suspected cases of DM. The average (±SD) for FPG was 94.86 ± 16.67 mg/dL. The mean ± SD for BMI was 21.79 ± 5.39 kg/m^2^. In terms of proportion, 67 (21.1%) of the study participants had BMI < 18.5 kg/m^2^, 193 (60.7%) were in the normal range (18.5-24.9 kg/m^2^), 48 (15.1%) were overweight, and 10 (3.1%) were obese. The proportion of participants with abnormal WC was 139 (43.6) with a disproportionate number being women. The mean ± SD for WC was 86.04 ± 11.25 cm. In addition, comparisons between males and females across disparate categories including educational level, habitual physical activity, frequency of traditional food intake, alcohol consumption, BMI, FPG, and WC exhibited a significant difference *p* < 0.001. Additional associations are in Table 1.

### 3.2. Frequency of Dyslipidemia and Isolated and Mixed Dyslipidemia

The prevalence of dyslipidemias was 70.5%. In terms of individual markers, the order was as follows: high TC (51.2%), LDL-C (43.7%), low HDL-C (28.2%), and TG (27.6%). The mean ± SD levels in mg/dL of TC, LDL-C, non-HDL-C, TG, HDL-C, TC/HDL-C, and TG/HDL-C were 202.2 ± 40.63, 125.95 ± 33.16, 151.72 ± 37.19, 129 ± 57.16, 50.48 ± 10.91, 4.11 ± 0.91, and 2.72 ± 1.49, respectively. In a female vs. male comparison, females had significantly higher mean values in all the lipid markers evaluated: higher TC (214.25 ± 39.37 vs. 192.16 ± 39.0 mg/dL in men, *p* < 0.001), higher LDL-C (134.21 ± 32.58 vs. 119.06 ± 32.13 mg/dL, *p* < 0.0012), higher HDL-C (52.87 ± 11.30 vs. 48.49 ± 10.18 mg/dL, *p* < 0.001), higher TG (136.34 ± 59.40 vs. 123.10 ± 54.66 mg/dL, *p* = 0.039), and higher non-HDL-C (161.38 ± 36.9 vs 143.67 ± 35.57, *p* < 0.001). Furthermore, HDL-C was the most common single lipid abnormality (35 (9.7%)). Separately, 60 (17.5%), 103 (21.6%), 35 (11.0%), and 16 (5.0%) had abnormalities in 1, 2, 3, and 4 lipid abnormalities. The coexistence of high TC+LDL-C was the most common mixed dyslipidemia. In participants with abnormality in three lipid markers, TC+TG+LDL-C was the most common abnormality (9.7%) (see Table 2).

### 3.3. Participants' Lipid Profiles as per the ATP III and Adult Treatment Panel III Risk Schema

The NCEP-ATP III guidelines were used to evaluate participants' atherosclerotic cardiovascular disease risk (ASCVD). Based on this scheme, 18.5%, 14.5%, 28.2%, and 12.9% were in high or very high-risk categories for TC, LDL-C, TG, and HDL-C, respectively. A significant difference between males and females was observed in all groupings assessed (see Table 3).

### 3.4. Factors Associated with Selected Dyslipidemias

The prevalence of elevated TC, LDL, TG, non-HDL-C, TC/HDL, and TG/HDL; the presence of at least one dyslipidemia; and low HDL-C have been stratified by specific covariates. Compared to males, females had significantly higher proportions in TC, LDL-C, non-HDL-C, TG, and dyslipidemia. Education level (mostly higher in the unemployed) was associated with high TC and low HDL-C, while the frequency of physical activity (higher in individuals having <1-hour daily physical activity) was associated with high LDL, non-HDL, TG, and TC/HDL ratio. Reduced frequency of traditional food consumption was associated with elevated TC, LDL-C, and non-HDL-C. Similarly, DBP ≥ 90 mmHg was associated with high TC, LDL-C, non-HDL-C, TG, and TC/HDL-C ratio and TG/HDL. At the same time, a positive diagnosis of hypertension was associated with elevated TC and low HDL. BMI and WC were significantly associated with all lipid markers evaluated (see Table 4).

### 3.5. Multivariate Analysis of the Factors Associated with Dyslipidemias

Adjusted logistic regression analysis on the relationship between specific lipid parameters and specific demographic and clinical parameters is presented in Table 5. In this analysis, TC ≥ 200 mg/dL was associated with age (aOR = 0.96, 95%CI = 0.918 − 0.995, *p* value = 0.027) and infrequent consumption of traditional foods (2-4 times a day (Ref) vs. once a day (aOR = 2.71, 95%CI = 0.78–9.33, *p* value = 0.100), 1-6 per week (aOR = 4.2, 95%CI = 2.6–8.2, *p* value < 0.001), and 1-3 months (aOR = 1.79, 95%CI = 1.00–3.21, *p* value = 0.024)), reduced in participants with DBP ≤ 90 mmHg (aOR = 0.583, 95%CI = 0.34–9.9, *p* value = 0.045), and increased with increasing FPG (aOR = 1.012, 95%CI = 1.0–1.03, *p* value = 0.036) and WC (aOR = 1.03, 95%CI = 1.0–1.05, *p* value = 0.032). Furthermore, LDL ≥ 130 mg/dL was associated with daily physical activity (<1 hour (aOR = 2.8, 95%CI = 1.05–7.75, *p* value = 0.001), 1-2 hours (aOR = 5.0, 95%CI = 1.77–14.4, *p* value = 0.017), and 2.3 hours (aOR = 2.3, 95%CI = 0.68–7.84, *p* value = 0.152)) and was higher in participants with infrequent consumption of traditional foods (2-4 times a day (Ref) vs. once a day (aOR = 2.0, 95%CI = 1.11 − 3.63, *p* value = 0.94), 1-6 per week (aOR = 2.95, 95%CI = 1.56–5.6, *p* value = 0.001), 1-3 months (aOR = 3.4, 95%CI = 1.01–11.6, *p* value = 0.012)). Additional associations in the model included reduced DBP (aOR = 0.6, 95%CI = 0.35–1.02, *p* value = 0.061) and WC (aOR = 1.03, 95%CI = 1.01–1.06, *p* value = 0.009). High TG was associated with DBP ≤ 90 mmHg (aOR = 0.43, 95%CI = 0.25–0.75, *p* value = 0.003), SBP ≥ 140 mmHg (aOR = 2.17, 95%CI = 1.14–4.1, *p* value = 0.003), elevated FPG (aOR = 1.02, 95%CI = 1.0–1.04, *p* value = 0.004), and WC (aOR = 1.06, 95%CI = 1.03–1.1, *p* value < 0.001). Low HDL-C was more frequent in females (aOR = 2.73, 95%CI = 1.58–4.7, *p* value < 0.001) and SBP > 140 mmHg (aOR = 2.19, 95%CI = 1.16–4.2, *p* value = 0.016) and was also associated with increasing BMI (aOR = 1.17, 95%CI = 1.08–1.03, *p* value < 0.001). See Table 5 for additional associations.

## 4. Discussion

At the end of the study, it was possible to establish the frequency of dyslipidemias and related associations. According to the data obtained, 225 (70.5%) (122 (54.2%) females vs. 103 (45.8%) in males, *p* value < 0.001) of the study participants had some type of dyslipidemia. These numbers are nearly 2-folds higher than the WHO 2008 estimate of 39% (40% women vs. 37% men) [21]. More importantly, some studies suggest that the prevalence of dyslipidemia in adults in Africa is between 15% and 50% [15, 22]. Due, in part, to differences in definition criteria, published prevalence estimates of dyslipidemia often vary. In addition, the scarcity of population-based studies on the burden of dyslipidemia in elderly populations in SSA limits the comparisons of our results. These notwithstanding, data from general populations have reported a prevalence of 67.3% in South Africa [23] and 71.3% in Uganda [24]. Unfortunately, the prevalence of dyslipidemia was nearly similar or even higher than what has been observed in some jurisdictions outside SSA—India (79%) [25] and China (39.9%) [26].

In terms of individual lipid and lipoprotein markers, the hierarchy of dominance was as follows: high TC (51.2%) > high LDL-C (43.7%) > low HDL-C (28.2%) > high TG (27.6%). The mean values of TC, LDL-C, non-HDL-C, TG, HDL-C, TC/HDL-C, and TG/HDL-C were also high with a significant disparity between males and females. Equally high prevalence, particularly of TC, has been observed in other jurisdictions in SSA: >70% TC levels in individuals aged >70 years in Nigeria [27]. When the mean values for lipids were analyzed, equally high magnitudes were observed. Another important observation was the fact that the high burden of dyslipidemia coexisted with a high burden of abdominal obesity (43.1%), dysglycemia (16%), hypertension (28.5%), and physical inactivity. It must be noted that the WHO threshold (≥110 mg/dL) was used for FPG; as such, the use of the American Diabetes Association (ADA) threshold of 100 mg/dL would have yielded higher proportions of dysglycemia. In a nutshell, a significant proportion of the elderly has elevated risk of CVD. Although the proportion of participants presenting with obesity was marginal, and most were in the normal range for BMI, normal BMI coexisted often with abnormal WC. The coexistence of low levels of general obesity with a very high burden of abdominal obesity (abnormality in WC) is in line with studies across broad population ranges in SSA [28].

Furthermore, controversies regarding some of the lipid markers evaluated in this study persist. For instance, questions persist about the role of TC as a general mortality risk among the elderly (≥85 years) with some investigators suggesting that the relationship may be positive, negative, or null [29]. A retrospective cohort study suggested that the elderly with the lowest quartile of TC (<175 mg/dL) and LDL-C (<100.4 mg/dL) were at higher risk of all-cause mortality [30]. Similarly, the importance of lipid ratios in evaluating CVD risk in geriatrics is underinvestigated. A similar situation exists with regard to mixed dyslipidemia. Regardless, it is our considered position that the high burden of CVD risk factors, particularly dyslipidemia, could partly explain the excess burden of CVD in the elderly in the setting.

In terms of sociodemographic characteristics, our results suggest that dyslipidemia and low HDL were independently associated with sex. Although this study was cross-sectional by design, the results were generally in keeping with those of the widely respected Framingham study [31]. In their analysis of a randomly selected population of elderly patients aged ≥65 years, the authors noted that dyslipidemia was 1.8 (95% CI: 1.1-3.1) times higher in females compared to males [32]. In general, some evidence suggests that women in LMIC subregions had higher TC than their counterparts in HIC. The contributory role of this difference to the global CVD risk of women in SSA has not been quantified, in part because of a lack of available historical data. Another lipid marker that had a strong association with sex was low HDL-C [17, 33]. The relationship is not unique and is well recognized in treatment guidelines such as NCEP-ATP which has a different threshold for men and women.

Regarding the analysis of physical activity and its relationship to disparate lipid disorders, our results suggest that low physical activity rate (particularly less than 1 hour of exercise per day) was independently associated with LDL − C ≥ 130 mg/dL, non − HDL ≥ 130 mg/dL, and total dyslipidemia. A strong relationship between low rates of physical activity and low HDL-C was also observed in the cross-tabulation analysis. The results uncovered in this analysis are broadly similar to what has been articulated in established literature [34]. In general, physical inactivity is associated with higher BP or cardiovascular deconditioning, worse cholesterol levels, poorer glucose metabolism, poorer mental health, and obesity in the elderly. Consequently, low physical activity was ranked as a leading risk factor for lost daily adjusted life years (DALY) in 2010 [5]. According to some investigators, the elimination of risky behaviors such as physical inactivity would make it possible to prevent at least 80% of CVD [6].

To a large extent, the data relating low physical activity to aberrant lipid concentrations is augmented by the analysis showing a strong association between type of work and multiple lipid parameters. In our analysis, manual workers were less likely to present with lipid abnormalities. Furthermore, women were more likely to be unemployed (particularly in manual work) or engage in physical activity and had worse cardiometabolic outcomes (Lipids, FPG, BMI, WC, DBP, and SBP). These associations are generally supported by pathophysiological data which suggest that physical activity has multiple physiological, immunological, and metabolic effects [35]. For instance, research suggests that vigorously contracting muscle can release large quantities of myokines with auto-, para-, and endocrine effects. The best-studied myokine is Interleukin-6 (IL-6) which, among a myriad of functions, stimulates *β*-cell proliferation, reduces stress induced *β*-cell apoptosis, and increases glucose transporter (GLUT4) translocation, thus, basal glucose uptake [36]. Moreover, the well-documented benefits of physical activity supersede molecular adaptations of working skeletal muscle and include improvement of coronary endothelial function, enhancement of muscle strength and power, and reduction in the aging process or sarcopenia. By and large, it is clear from our analysis that physical inactivity and sedentary behavior differ by sex with women on the high-risk side. In a way, physical inactivity and sedentarism may partially explain the observed disparities in lipid health or other metabolic abnormalities observed in this population. The possibility has far-reaching implications for intervention efforts in this setting and requires further elucidation. Therefore, and to reiterate a general guideline, improving physical activity among the elderly population represents a cheap, realistic, and cost-effective pathway for improving lipid health in this population.

An analysis of the frequency of consumption of traditional food and its association with specific lipid biomarkers was also attempted. Interestingly, infrequent consumption of traditional foods was associated with aberrant TC, LDL, and non-HDL-C concentrations. The observed relationship may be linked to the nature of traditional cuisine in the country. In general, the traditional Eritrean cuisine is low in energy density and high in fiber content and deemphasizes the heavy use of oils or fats. Reduced intake of energy-dense micronutrient-poor foods and high dietary intake of fiber is recommended by WHO as lifestyle targets for reducing obesity. By and large, the observed relationships are important in multiple respects. First and foremost, investigators have attempted to formulate culturally adopted diets which may be used by high-risk segments of the population to ameliorate CVD risk. Accordingly, the data suggests that in Eritrea, a country with a minuscule number of dieticians and where such efforts have not been undertaken; the solution or starting point appears to be a renewed emphasis on traditional cuisines. Second, the data confirms the much-touted possibility that dietary transition or westernization of diets among elderly communities living in urban centers in SSA is aggravating CVD burden. Despite the preliminary nature of these results, they point to an intriguing area for future inquiry—studies on the relationship between consumption of traditional foods and impact on lipid health or health in general.

In a separate analysis, we demonstrated a strong positive cross-sectional association between BMI, WC, and abnormalities in all the lipid markers. A linear-by-linear association was also prominent. However, associations between BMI and multiple lipid disorders were mostly attenuated in the adjusted logistic regression analysis. Interestingly, a strong association between WC (a surrogate marker of visceral adiposity), FPG, elevated DBP, and multiple dyslipidemias persisted in the multivariable logistic regression analysis. These associations have been observed in younger cohorts [25, 36]. From a more practical perspective, computed tomography (CT) studies have demonstrated that visceral adiposity is more closely related to lipid, glucose, and blood pressure abnormalities [37]. The copresence of these abnormalities, the metabolic syndrome (also known as “the deadly quartet for CVD”), has therefore been explained by a shared pathophysiological process with abnormality in WC as the trigger [38]. Importantly, studies have shown that reduction of visceral fat ameliorates the “deadly quartet” simultaneously. To circle back to our original point, WC and not BMI ≥ 25 kg/m^2^ is a more important driver of CVD in the elderly population in this setting.

Although the importance of WC is well documented and appears to play a prominent role in this setting, its use is not well established in Eritrea. This cheap-to-measure marker is rarely recorded by clinicians, and awareness regarding the dangers of high WC values is low. Instead, the use of BMI, even in the elderly, prevails. About the latter issue, we have to note that although BMI has a good positive correlation with total adiposity and with morbimortality from many diseases [39], the exclusive use of BMI in the elderly has been questioned owing to its poor correlation with visceral adiposity [40]. Higher central adiposity at normal BMI levels is a common presentation in the elderly particularly in those with some degree of sarcopenia. However, we have to concede that the absence of WC cut-off for the region militates against the widespread use of WC. This notwithsatnding, the simultaneous use of BMI and WC, a prescription in many treatment guidelines, should be encouraged in this setting.

### 4.1. Study Limitations and Strength

Understanding the burden, distribution, and factors associated with dyslipidemia in elderly populations is of paramount importance. The importance of such studies is magnified in countries in SSA where very little is known about the health of the elderly populations. This has undermined population-level efforts targeted at prevention or health education. In this regard, it is our opinion that this study, although preliminary, can contribute substantially to a better understanding of the burden of dyslipidemias and associated risk factors in this jurisdiction. This aside, some limitations must be mentioned. The sample represents a group of elderly participants working or residing within specific residential estates in Asmara, Eritrea. As such, it can be argued that the sample was not fully randomized. Nevertheless, this mode of data acquisition is superior to outpatients' data—a method that is prominent in most studies from the region. Importantly, and for the most part, the subjects presented with sociodemographic and clinical characteristics that were broadly similar to the general elderly population. Additional methodological issues that can undermine our results were the cross-sectional nature of the study and the fact that subclinical CVD events or comorbidities were not evaluated. Importantly, the use of hemoglobin A_1C_ to verify cases of DM or more granular characterization of physical activity and traditional food regimens could have added value to the study. For example, physical activity measurement instruments generally omit domestic activities such as cooking, cleaning, and childcare in physical activity computations. This was the case in this study. Lastly, miscoding, clerical errors, and unverifiable responses by respondents may also be limiting. Indeed, older adults are prone to forgetfulness and often overestimate their level of physical activity.

## 5. Conclusions

Several important findings emerged from this study. First, a large proportion of the study participants had some type of dyslipidemia (70.5%). All the study participants were not on any medication, and all were unaware of their condition. The most dominant dyslipidemia was high TC (51.2%), followed by low LDL-C (43.7%), low HDL-C (28.2%), and high TG (27.6%). Regarding NCEP-ATP III risk strata, 18.5%, 14.5%, 28.2%, and 12.9% were in high or very high-risk categories for TC, LDL-C, TG, and HDL-C, respectively. The most predominant mixed dyslipidemia was the TC+LDL-C combination. At the same time, 60 (17.5%), 103(21.6%), 35 (11.0%), and 16 (5.0%) had abnormalities in 1, 2, 3, and 4 lipid abnormalities. Based on the study results, it is our opinion that a large proportion of the study is at risk of developing or aggravating preexisting CVD. Women were disproportionately affected across most of these categories. Furthermore, stepwise multivariate modeling demonstrated that the frequency of dyslipidemia was associated with sex, daily physical activity, increasing FPG, and BMI. Apart from the poor lipid health, a large proportion of the study participants had dysglycemia and elevated BP or hypertension. The proportion of the study participants presenting with abdominal obesity, a powerful surrogate marker of CVD, was also high. Taken together, these observations call for concerted effort directed at scaling up early recognition and treatment, including optimal pharmacological and nonpharmacological therapy at all levels of care.

## Figures and Tables

**Table 1:** Demographic, anthropometric, and clinical characteristics of elderly participants in Asmara, Eritrea.

Covariate	Presence of dyslipidemia *N* (%)		*p* value, chi-square	Total
	Female	Male		
*Age (years)*				
>60–65years	55 (55.6)	44 (44.4)	0.095 (6.38)	99 (31.0)
>65–70 years	40 (43.0)	53 (57.0)		93 (29.2)
>70–75 years	26 (41.3)	37 (58.7)		63 (19.7)
>75 years	24 (37.5)	37 (58.70)		67 (20.1)
*Educational level*				
No education	83 (78.3)	23 (21.7)	<0.001 (76.32)	106 (33.2)
Elementary	38 (38.4)	61 (61.6)		99 (31.0)
Secondary	14 (23.7)	45 (76.3)		59 (18.5)
Senior	7 (15.9)	37 (84.1)		44 (13.8)
Tertiary	3 (27.3)	8 (72.7)		11 (3.4)
*Daily physical activity*				
<1 hour	122 (62.6)	73 (37.4)	<0.001 (72.34)	195 (61.3)
1-2 hours	20 (33.3)	40 (66.7)		60 (18.9)
2-3 hours	0 (0.0)	27 (100)		27 (8.5)
>3 hours	2 (5.6)	34 (94.4)		36 (11.3)
*Employment status*				
Unemployed	117 (98.3)	2 (1.7)	<0.001 (215.81)	119 (37.3)
Office work	8 (25.0)	24 (75.0)		32 (10.0)
Manual work	20 (11.9)	148 (88.1)		168 (52.7)
*Traditional food (frequency)*				
1-3 per month	11 (85.3)	2 (14.7)	<0.001 (40.61)	13 (4.1)
1-6 per week	43 (64.2)	24 (35.8)		67 (21.1)
Once a day	47 (55.3)	38 (44.7)		85 (26.7)
2-4 times a day	43 (28.1)	110 (71.9)		153 (48.1)
*Alcohol consumption*				
No	114 (57.6)	84 (42.4)	<0.001 (30.93)	198 (62.1)
Yes	31 (25.6)	90 (74.04)		121 (37.9)
*Diastolic blood pressure*				
<90 mmHg	98 (46.2)	114 (53.8)	0.722 (0.152)	212 (66.5)
>90 mmHg	47 (43.9)	60 (56.1)		107 (33.5)
*Systolic blood pressure*				
<140 mmHg	109 (48.0)	118 (52.0)	0.172 (2.09)	227 (71.2)
>140 mmHg	36 (39.1)	56 (60.9)		92 (28.8)
*Presence of hypertension*				
Yes	46 (50.5)	45 (49.5)	0.152 (1.33)	91 (28.5)
No	99 (43.4)	129 (56.6)		228 (71.5)
*Fasting plasma glucose (FPG)*				
Normal	110 (41.0)	158 (59.0)	<0.001 (14.45)	268 (84.0)
Prediabetes	20 (62.5)	12 (37.5)		32 (10.0)
Suspected DM	15 (78.9)	4 (21.1)		19 (6.0)
*Body mass index (BMI) (kg/m^2^)*				
<18.5	27 (40.3)	40 (59.7)	<0.001 (20.32)	67 (21.1)
18.5-24.9	76 (39.4)	117 (60.6)		193 (60.7)
25-29.9	32 (66.7)	16 (33.3)		48 (15.1)
>30	9 (90.0)	1 (10.0)		10 (3.1)
*Waist circumference (WC)*				
Normal	42 (23.3)	138 (76.7)	<0.001 (79.30)	180 (56.4)
Abnormal	103 (74.1)	36 (25.9)		139 (43.1)

**Table 2:** Frequency of dyslipidemia and isolated and mixed dyslipidemia.

Lipid abnormality	Female *N* (%)	Male *N* (%)	Difference (%)	Total frequency *N* (%)
No lipid abnormality	23 (24.5)	71 (75.5)	-51.0	94 (29.5)
Isolated dyslipidemias				
One abnormality				
TC	6 (60.0)	4 (40.0)	20.0	10 (3.1)
TG	3 (27.3)	8 (72.7)	-45.4	11 (3.4)
HDL-C	19 (54.3)	16 (45.7)	8.60	35 (9.7)
LDL-C	1 (25.0)	3 (75.0)	-50.0	4 (1.3)
Total				60 (17.5)
Mixed dyslipidemias				
Two abnormalities				
TG+low-HDL-C	8 (53.3)	7 (46.7)	6.6	15 (4.7)
LDL+low-HL-C	6 (75.0)	2 (25.0)	50.0	8 (2.5)
TC+TG	5 (45.5)	6 (54.5)	-9.0	11 (3.4)
TC+LDL-C	34 (49.3)	35 (50.7)	-1.40	69 (21.6)
Total				103 (21.6)
Three abnormalities				
TG+TC+HDL-C	4 (100)	0 (0)	100	4 (1.3)
TC+TG+LDL	13 (41.9)	18 (58.1)	-16.20	31 (9.7)
Total				35 (11.0)
Four abnormalities				
TG+TC+HDL-C+LDL-C	14 (87.5)	2 (12.5)	75.0	16 (5.0)
Dyslipidemia	122 (54.2)	103 (45.8)	8.40	225 (70.5)

LDL: low-density lipoproteins; HDL-C: high-density lipoproteins; TG: triglycerides; TC: total cholesterol.

**Table 3:** NCEP-ATP III based characterization of lipid disorders in the elderly in Asmara, Eritrea.

ATP III classification	Sex		*p* value, chi-square	Percentage (%)
	Female	Male		
*Total cholesterol (mg/dL)*				
Optimal serum concentration (< 200)	54 (34.0)	105 (66.0)	21.38 0.001	159 (49.8)
Borderline (200-239)	51 (50.5)	50 (49.5)		101 (31.7)
High-risk serum conc. (≥240)	40 (67.8)	19 (32.2)		59 (18.5)
*Triglyceride (mg/dL)*				
Normal (<150)	98 (42.4)	133 (57.6)	4.87 0.087	231 (72.4)
Borderline high (150-199)	22 (46.8)	25 (53.2)		47 (14.7)
High (≥200)	25 (61.0)	16 (39.0)		41 (12.9)
*LDL-C (mg/dL)*				
Optimal (<100)	17 (27.4)	45 (72.6)	16.60 0.002	62 (19.4)
Near-optimal (100-129)	51 (43.2)	67 (56.8)		118 (37.0)
Borderline high (130-159)	47 (50.5)	46 (49.5)		93 (29.2)
High (160-189)	22 (64.7)	12 (35.3)		34 (10.7)
Very high (≥190)	8 (66.7)	4 (33.3)		12 (3.8)
*HDL-C*				
Optimal (≥60)	35 (64.8)	19 (35.2)	44.50 0.001	54 (17.1)
Borderline men (40-59) and women (50-59)	50 (28.9)	123 (71.1)		173 (54.7)
High risk (<40 men) and (<50 women)	60 (67.4)	29 (32.6)		89 (28.2)

Abbreviation: LDL: low-density lipoproteins; HDL-C: high-density lipoproteins; Conc.: concentration.

**Table 4:** Single and mixed dyslipidemia and some associated factors in the elderly in Asmara, Eritrea.

Covariate	TC	LDL-C	Non-HDL-C	TG	HDL	TC/HDL	TG/HDL	Dyslipidemia
	≥200 mg/dL	≥130 mg/dL	≥130	≥150 mg/dL	Low	≥3.5	≥4	
*Sex*								
Female	**85 (58.6)**	**77 (53.1)**	**118 (81.4)**	**47 (32.4)**	**60 (41.4)**	30 (20.7)	35 (24.1)	**122 (84.1)**
Male	**67 (38.5)**	**62 (35.6)**	**105 (60.3)**	**41 (23.6)**	**29 (16.7)**	28 (16.1)	41 (23.6)	**103 (59.2)**
*Age (years)*								
>60–65years	57 (57.6)	44 (44.4)	72 (72.7)	32 (32.3)	34 (34.3)	20 (20.2)	27 (27.3)	80 (80.8)
>65–70 years	39 (41.9)	39 (41.9)	61 (65.6)	22 (23.7)	23 (24.7)	15 (16.1)	19 (20.4)	59 (63.4)
>70–75 years	25 (39.7)	27 (42.9)	47 (74.6)	22 (34.9)	16 (25.4)	11 (17.5)	16 (25.4)	43 (68.3)
>75 years	31 (48.4)	29 (45.3)	43 (67.2)	12 (18.8)	16 (25.0)	12 (18.8)	14 (21.9)	43 (67.2)
*Educational level*								
No education	**60 (56.6)**	54 (50.9)	82 (77.4)	31 (29.2)	**40 (37.7)**	22 (20.8)	26 (24.5)	86 (81.1)
Elementary	**39 (39.4)**	39 (39.4)	65 (65.7)	23 (23.2)	**26 (26.3)**	14 (14.1)	21 (21.2)	63 (63.6)
Junior	**28 (47.5)**	22 (37.3)	36 (61.0)	17 (28.8)	**13 (22.0)**	12 (20.3)	15 (25.4)	39 (66.1)
Senior	**23 (52.3)**	21 (47.7)	33 (75.0)	15 (34.1)	**5 (11.4)**	7 (15.9)	12 (27.3)	30 (68.2)
Tertiary	**2 (18.2)**	3 (27.3)	7 (63.6)	2 (18.2)	**5 (45.5)**	3 (27.3)	2 (18.2)	7 (63.6)
*Daily physical activity*								
<1 hour	99 (50.8)	**88 (45.1)**	**141 (72.3)**	58 (29.7)	**65 (33.3)**	40 (20.5)	46 (23.6)	**147 (75.4)**
1-2 hours	30 (50.0)	**33 (55.0)**	**43 (71.7)**	15 (25.0)	**16 (26.7)**	9 (15.0)	14 (23.3)	**45 (75.0)**
2- 3 hours	12 (44.4)	**10 (37.0)**	**21 (77.8)**	4 (14.8)	**4 (14.8)**	4 (14.8)	4 (14.8)	**16 (59.3)**
>3 hours	10 (27.8)	**7 (19.4)**	**17 (47.2)**	11 (30.6)	**4 (11.1)** ^∗^	5 (13.9)	12 (33.3)	**16 (44.4)**
*Employment status*								
Unemployed	**70 (58.8)**	**64 (53.8)**	**98 (82.4)**	**38 (31.9)**	**47 (39.5)**	23 (19.3)	27 (22.7)	**99 (83.2)**
Office work	**16 (50.0)**	**16 (50.0)**	**24 (75.0)**	**13 (40.6)**	**6 (18.8)**	4 (12.5)	11 (34.4)	**24 (75.0)**
Manual work	**66 (39.3)** ^∗^	**59 (35.1)** ^∗^	**101 (60.1)**	**37 (22.0)**	**36 (21.4)**	31 (18.5)	38 (22.6)	**102 (60.7)** ^∗^
*Traditional food (frequency)*								
1-3 per month	**8 (61.5)**	**8 (61.5)**	**12 (92.3)**	4 (30.8)	7 (53.8)	3 (23.1)	4 (30.8)	**11 (84.6)**
1-6 per week	**46 (68.7)**	**39 (58.2)**	**56 (83.6)**	23 (34.3)	19 (28.4)	14 (20.9)	18 (26.9)	**54 (80.6)**
Once a day	**45 (52.9)**	**43 (50.6)**	**66 (77.6)**	25 (29.4)	23 (27.1)	14 (16.5)	20 (23.50	**65 (76.5)**
2-4 times a day	**53 (34.6)**	**49 (32.0)**	**89 (58.2)**	35 (22.9)	39 (25.5)	27 (17.6)	33 (21.6)	**94 (61.4)**
*Alcohol consumption*								
No	**109 (55.1)**	**101 (51.0)**	**157 (79.3)**	59 (29.8)	59 (29.8)	44 (22.2)	49 (24.7)	**151 (76.3)**
Yes	**43 (35.5)**	**38 (31.4)**	**66 (54.5)**	29 (24.0)	30 (24.8)	14 (11.6)	27 (22.3)	**74 (61.2)**
*Diastolic blood pressure*								
<90 mmHg	**42 (19.8)**	**83 (39.2)**	**139 (65.6)**	**89 (42.0)**	61 (28.8)	**31 (14.6)**	**36 (17.0)**	**142 (67.0)**
>90 mmHg	**46 (43.0)**	**56 (52.3)**	**84 (78.5)**	**63 (58.9)**	28 (26.2)	**27 (25.2)**	**40 (37.4)**	**83 (77.6)**
*Systolic blood pressure*								
<140 mmHg	**99 (43.6)**	92 (40.5)	152 (67.0)	60 (26.4)	**72 (31.7)**	40 (17.6)	54 (23.8)	158 (69.6)
>140 mmHg	**53 (57.6)**	47 (51.1)	71 (77.2)	28 (30.4)	**17 (18.5)**	18 (19.6)	22 (23.9)	225 (70.5)
*Presence of hypertension*								
Yes	**53 (58.2)**	47 (51.6)	71 (78.0)	**36 (39.6)**	27 (29.7)	22 (24.2)	**31 (34.1)**	70 (76.9)
No	**99 (43.4)**	92 (40.4)	152 (66.7)	**52 (22.8)**	62 (27.2)	36 (15.8)	**45 (19.7)**	155 (68.0)
*Fasting plasma glucose (FPG)*								
Normal	121 (45.1)	111 (41.4)	**175 (66.4)**	**60 (22.4)**	**69 (25.7)**	**41 (15.3)**	**52 (19.4)**	**179 (66.8)**
Prediabetes	20 (62.5)	16 (50.0)	**28 (87.5)**	**16 (50.0)**	**12 (37.5)**	**11 (34.4)**	**14 (43.8)**	**29 (90.6)**
Suspected DM	11 (57.9)	12 (63.2)	**17 (89.5)**	**12 (63.2)** ^∗^	**8 (42.1)** ^∗^	**6 (31.6)**	**10 (52.6)** ^∗^	**17 (89.5)** ^∗^
*Body mass index (BMI) (kg/m^2^)*								
<18.5	**23 (34.3)**	**20 (29.9)**	**33 (49.3)**	**5 (7.5)**	**13 (19.4)**	**2 (3.0)**	**2 (3.0)**	**36 (53.7)**
18.5-24.9	**95 (49.2)**	**85 (44.0)**	**140 (72.5)**	**54 (28.0)**	**47 (24.4)**	**37 (19.2)**	**48 (24.9)**	**136 (70.5)**
25-29.9	**26 (54.2)**	**25 (52.1)**	**40 (83.3)**	**23 (47.9)**	**20 (41.7)**	**11 (22.9)**	**18 (37.5)**	**42 (87.5)**
>30	**7 (70.0)** ^∗^	**8 (80.0)** ^∗^	**9 (90.0)** ^∗^	**6 (60.0)** ^∗^	**8 (80.0)** ^∗^	**8 (80.0)** ^∗^	**8 (80.0)** ^∗^	**10 (100.0)** ^∗^
*Waist circumference (WC)*								
Normal	**72 (40.0)**	**67 (37.2)**	**109 (60.6)**	**31 (17.2)**	**33 (18.3)**	**21 (11.7)**	**29 (16.1)**	**109 (60.6)**
Abnormal	**80 (57.6)**	**72 (51.8)**	**114 (82.0)**	**57 (41.0)**	**56 (40.3)**	**37 (26.6)**	**47 (33.8)**	**116 (83.5)**

All the presented cross-tabulations are significant at *p* < 0.05. Bold letters: *p* < 0.05. ^∗^Linear-by-linear association. The proportion of the population with lipid disorders considered in the analysis. HDL: high-density lipoprotein; LDL: low-density lipoprotein; TG: triglycerides; TC: total cholesterol; non-HDL: non-high-density lipoprotein cholesterol.

**Table 5:** Factors associated with dyslipidemia from a univariate logistic regression model.

Variables	TC ≥ 200 mg/dL aOR (95% CI)	LDL ≥ 130 mg/dL aOR (95% CI)	Non-HDL mg/dL aOR (95% CI)	TG ≥ 150 mg/dL aOR (95% CI)	HDL low aOR (95% CI)	TC/HDL ≥ 5 aOR (95% CI)	TG/HDL > 4 aOR (95% CI)	Dyslipidemia aOR (95% CI)
*Sex*								
Male					**1**			1
Female					**2.73 (1.58-4.7)**			2.6 (1.1-6.1)
*p* value					**<0.001**			**0.017**
*Age (years)*								
	0.97 (0.93-1.01)							0.96 (0.92-1.0)
*p* value	0.027							0.087
*Daily physical activity*								
<1 hour		**2.8 (1.05-7.15)**	**4.5 (1.28-15.7)**					**2.6 (1.1-6.1)**
*p* value		**0.015**						**0.029**
1-2 hours		**5.0 (1.77-14.4)**	2.36 (0.86-6.4)					**3.2 (1.24-8.5)**
*p* value		**0.001**						**0.016**
2-3 hours		2.3 (0.68-7.84)	1.59 (0.65-3.89)					2.0 (0.7-5.8)
*p* value		0.017						0.192
>3 hours		**1**	**1**					1
*p* value		**0.010**						
*Employment status*								
Unemployed			**2.23 (1.1-4.5)**					
*p* value								
Office work			1.8 (0.67-4.85)					
*p* value								
Manual work			**1**					
*p* value								
*Traditional food (consumption frequency)*								
2-4 times a day	1	**1**	**1**					
*p* value	**<0.001**	**0.004**						
Once a day	2.7 (0.78-9.33)	**2.0 (1.11-3.63)**	7.4 (0.8-26)					
*p* value	**0.0100**	**0.094**						
1-6 per week	**4.2 (2.6-8.2)**	**2.95 (1.56-5.6)**	**3.19 (1.4-7.2)**					
*p* value	**<0.001**	**0.001**						
1-3 months	**1.79 (1.0-3.21)**	**3.4 (1.01-11.6)**	**2.38 (1.19-4.78)**					
	**0.039**	**0.012**						
*Diastolic blood pressure*								
>90 mmHg	1	**1**	**1**	**1**			1	
<90 mmHg	0.583 (0.34-9.9)	**0.6 (0.35-1.02)**	0.53 (0.28-1.03**)**	**0.43 (0.25-0.75)**			**0.37 (0.19-0.69)**	
*p* value	0.039	**0.061**	**0.059**	0.003			**0.002**	
*Systolic blood pressure*								
<140 mmHg				**1**	1		1	
>140 mmHg				**2.17 (1.14-4.1)**	2.19 (1.16-4.17)		**2.12 (1.07-4.24)**	
*p* value				**<0.001**	<0.001		**0.033**	
FPG	**1.012 (1.0-1.03)**	**1.01 (1.0-1.03)**	**1.03 (1.01-1.06)**	**1.02 (1.0-1.04)**			1.02 (1.00-1.04)	**1.02 (1.0-1.04)**
*p* value	**0.036**		**0.003**	**0.004**			0.019	**0.039**
BMI (kg/m^2^)					**1.17 (1.08-1.3)**			**1.19 (1.09-1.3)**
*p* value					<0.001			**<0.001**
Waist	**1.03 (1.0-1.05)**	**1.03 (1.01-1.06)**	**1.05 (1.02-1.08)**	**1.06 (1.03-1.1)**		**1.07 (1.04-1.1)**	**1.07 (1.04-1.10)**	
*p* value	**0.032**	**0.009**	**<0.001**	<0.001		**<0.001**	**<0.001**	

All the presented aOR are significant at *p* value < 0.05. Bold letters: *p* value < 0.05. Proportion of the population with lipid disorders considered in the analysis. HDL: high-density lipoprotein; LDL: low-density lipoprotein; TG: triglycerides; TC: total cholesterol; non-HDL: non-high-density lipoprotein cholesterol; FPG: fasting plasma glucose; BMI: body mass index; aOR: adjusted odds ratios; CI: confidence interval.*p*value = 0.05. Note that age, FPG, BMI, and WC were modeled as continuous variables.

## Data Availability

The dataset supporting the conclusions of this article is available from the corresponding author on reasonable request.
